# Decoding the PTTG family’s contribution to LUAD pathogenesis: a comprehensive study on expression, epigenetics, and therapeutic interventions

**DOI:** 10.1186/s41065-025-00545-x

**Published:** 2025-08-28

**Authors:** Jinna Di, Li Tian, Fan Yan, Zhang Zhe, Cai Lin, Liu Jingyu

**Affiliations:** 1 Department of Respiratory and Critical Care Medicine, The Third Affiliated Hospital of Jinzhou Medical University, No. 2 Section 5 Heping Road, Linghe District, Jinzhou, 121000 Liaoning China; 2 Department of Respiratory and Critical Care Medicine, The First Affiliated Hospital of Jinzhou Medical University, No. 2 Section 5 Guta Road, Renmin District, Jinzhou, 121000 Liaoning China

**Keywords:** PTTG genes, LUAD, Biomarker, Treatment, Gene expression, Mutations

## Abstract

**Background:**

Lung adenocarcinoma (LUAD) stands as a prevalent malignancy, yet its pathology remains incompletely comprehended.

**Methods:**

In this comprehensive study, we explored the roles of the pituitary tumor-transforming gene (PTTG) family, including PTTG1, PTTG2, and the pseudogene PTTG3P in lung adenocarcinoma (LUAD). Employing a multi-faceted approach, we conducted in-depth analyses using clinical samples and expression datasets.

**Results:**

Our findings revealed a significant up-regulation of PTTG family genes in LUAD cell lines and tissue samples compared to adjacent normal controls, suggesting their potential as diagnostic biomarkers. Through promoter methylation and mutational analyses, we uncovered regulatory mechanisms influencing PTTG gene expression. The exploration of the PTTG family’s impact on LUAD patient survival demonstrated their association with adverse outcomes, emphasizing their potential prognostic relevance. Moreover, functional assays demonstrated that the knockdown of PTTG1 and PTTG2 genes resulted in the reduction of cell proliferation, colony formation, and cell migration abilities in A549 and H1975 LUAD cells. Furthermore, our investigation extended to therapeutic avenues, where we identified Calcitriol as a potential drug within the DrugBank database to down-regulate PTTG genes. Molecular docking analyses provided insights into the strong interaction between Calcitriol and PTTG1/PTTG2 proteins, laying the groundwork for further exploration of Calcitriol in LUAD treatment.

**Conclusion:**

In conclusion, this study contributes a comprehensive understanding of the PTTG family’s involvement in LUAD, shedding light on their diagnostic, prognostic, and therapeutic implications.

**Supplementary Information:**

The online version contains supplementary material available at 10.1186/s41065-025-00545-x.

## Introduction

Lung adenocarcinoma (LUAD), a prevalent and life-threatening malignancy, remains a significant global health concern, necessitating continuous efforts to unravel its underlying molecular mechanisms for improved therapeutic strategies [1–3]. Among the myriad of genes implicated in cancer progression, the Pituitary Tumor-Transforming Gene (PTTG) family, consisting of tumor-transforming 1 (PTTG1), pituitary tumor-transforming 2 (PTTG2), and pituitary tumor-transforming 3P (PTTG3P) genes, has emerged as a compelling focus of investigation due to its intricate involvement in various cellular processes [4]. PTTG1 shares homology with both PTTG2 and PTTG3P [5, 6], and research indicates its up-regulation in various endocrine-related malignancies through investigations conducted using both in silico and molecular experiments [7]. While there is limited understanding of the biological roles of PTTG2, this protein, along with its associated counterpart, PTTG3P, has been implicated in the development of numerous human cancers [8].

Recent studies indicate the involvement of PTTG1 and PTTG2 in the carcinogenic process [9–11], while PTTG3P, characterized by its intron-free structure and considerable homology to both PTTG1 and PTTG2, is also implicated in certain cellular processes [5]. The PTTG family exhibits overexpression in various cancer types, including but not limited to gastric cancer, kidney cancer, pancreatic cancer, breast cancer, liver cancer, and esophageal cancer [12–15]. The dysregulation of PTTG1 amplifies the proliferation, invasion, and metastasis of tumor cells while concurrently suppressing apoptosis [16–18]. PTTG2 and PTTG3P, being homologous to PTTG1 [19], have roles not fully elucidated, but their confirmed association with human cancer development is acknowledged. For instance, an earlier study demonstrated a significant up-regulation of PTTG2 in glioblastoma, establishing its overexpression as a promoter of glioblastoma cell proliferation and invasion [20]. Another study demonstrated that PTTG3P can augment the in vitro proliferation and invasion of gastric cancer (GC), serving as an indicator of an unfavorable prognosis [21]. One more study observed a significant elevation in PTTG1 mRNA expression in four out of the six human GC cell lines compared to their counterparts with lower counts, aligning with the consistent data of mRNA expression [22].

However, the specific contributions and differential functions of individual PTTG family members—particularly PTTG1 and PTTG3P—in LUAD remain largely unexplored. This study uniquely investigates the distinct biological and clinical implications of PTTG1, PTTG2, and PTTG3P in LUAD progression, with an emphasis on their expression patterns, prognostic relevance, and molecular regulation, including promoter methylation and mutational events. Notably, we distinguish the oncogenic functions of PTTG1 and PTTG2 through knockdown experiments in LUAD cell lines, while also examining the pseudogene PTTG3P as a potential regulatory non-coding RNA. Furthermore, this study preliminarily identifies Calcitriol as a potential therapeutic candidate for targeting PTTG1 and PTTG2 in LUAD, based on molecular docking analyses. However, these findings require further experimental validation to confirm pharmacological efficacy.

This study employs in silico and molecular experiments to elucidate the relationship between PTTGs and various aspects of LUAD, encompassing gene expression, promoter methylation analysis, mutational analysis, gene enrichment, and molecular docking analysis etc. The findings highlight the substantial impact of PTTGs on the initiation and progression of LUAD, offering new avenues for further research into the disease, providing insights into its intricate pathophysiology, and suggesting potential therapeutic interventions.

## Methodology

### Cell culture

In this study, 10 LUAD cell lines and 10 normal lung epithelial cell lines were purchased from American Type Culture Collection (ATCC). The LUAD cell lines included: A549, NCI-H1975, SK-LU-1, NCI-H2228, NCI-H2122, NCI-H1651, NCI-H1563, NCI-H2342, NCI-H2087, and NCI-H1568. The normal lung epithelial cell lines included: HSAEC1-KT, HSAEC, HCC4006, NuLi-1, BEAS-2B, NL20-TA, NL20, CuFi-1, and HBEC3-KT. Upon receipt, the cells were immediately cultured under optimal conditions specified by the ATCC guidelines. LUAD cell lines were grown in RPMI-1640 medium supplemented with 10% fetal bovine serum (FBS), 1% penicillin-streptomycin, and incubated at 37 °C in a humidified atmosphere with 5% CO_2_. Similarly, normal lung epithelial cell lines were cultured in DMEM (Dulbecco’s Modified Eagle Medium) with 10% FBS and 1% penicillin-streptomycin, also under the same temperature and CO_2_ conditions. The cells were passaged regularly when they reached approximately 80% confluence. Cell line authentication was carried out following the recommended procedures from ATCC to ensure the identity and integrity of the cell cultures used in the experiments.

### Experimental setup

For all experiments, cells were seeded at a density of 1 × 10⁵ cells per well in a 6-well plate and allowed to adhere overnight. Following the attachment period, the cells were treated with specific compounds or were left untreated to serve as control groups. Various assays, including proliferation, wound healing, and gene expression analyses, were performed according to the aims of the study.

### Maintenance and subculture

To maintain cell viability and prevent contamination, regular subculturing was carried out according to standard cell culture procedures. The cells were maintained at a low passage number (between passages 5 and 15) to avoid genetic drift. The growth medium was changed every 2 to 3 days, and cells were observed under an inverted microscope to monitor for any signs of contamination or morphological changes.

### Quality control

Cell authentication and mycoplasma testing were performed periodically to ensure the validity of the results and to maintain the authenticity of the cell lines. Additionally, cell morphology and growth characteristics were monitored to verify that the cells maintained typical characteristics for each cell line.

### Nucleic acid extraction

DNA extraction from the LUAD and control tissue samples was accomplished through the organic method [23–25], a well-established procedure recognized for its efficiency in yielding high-quality genomic material. Simultaneously, RNA extraction was carried out using the TRIzol method [26–28], a robust technique renowned for its efficacy in isolating intact RNA from diverse biological samples. The integrity and purity of the extracted nucleic acids, both DNA and RNA, were meticulously assessed through the 280/260 ratio method [29, 30]. This quality control step ensures that the obtained genetic material is of high purity, free from contaminants, and suitable for downstream molecular analyses.

### RT-qPCR analysis

The extracted RNA was reverse transcribed into complementary DNA (cDNA) using a Reverse Transcription kit (TOYOBO, Shanghai, China). Following this, RT-qPCR was performed with the SYBR Green PCR mix (Thermo Fisher Scientific, Waltham, USA) on the ABI 7900HT FAST Real-Time PCR System (Applied Biosystems, Foster City, CA, USA). GAPDH gene expression was used as the internal control for normalization, and relative mRNA expression levels were calculated using the 2^−ΔΔCT^ method. The primers used for amplifying GAPDH and PTTG family genes are listed below.

GAPDH-F 5’-ACCCACTCCTCCACCTTTGAC-3’,

GAPDH-R 5’-CTGTTGCTGTAGCCAAATTCG-3’

PTTG1-F: 5’-GCTTTGGGAACTGTCAACAGAGC-3’

PTTG1-R: 5’-CTGGATAGGCATCATCTGAGGC-3’

PTTG2-F: 5’-CTTTGGGCACTGTCAACAGAGC-3’

PTTG2-R: 5’-TCTGGATAGGCGTCATCTGAGG-3’

PTTG3P-F: 5’-CTGCCTGAAGAGCACCAGATTG-3’

PTTG3P-R: 5’-CATGGTGGAGAGGGCATCTTCA-3’

### Bisulfite sequencing

Bisulfite sequencing for targeted sequencing of the cell lines was performed by the Beijing Genomic Institute (BGI), China.

### Expression validation of the PTTG family genes across additional datasets

Our study extensively utilized UALCAN, GEPIA2 and the GEO database for the rigorous validation of PTTG family gene expression in LUAD patients. UALCAN, a user-friendly and comprehensive cancer data analysis platform, facilitated the in-depth exploration of cancer genomics data, aiding our investigation into the intricate expression patterns of PTTG genes in TCGA LUAD patients [31]. This platform primarily utilizes data from TCGA, a comprehensive resource that includes a large number of cancer samples with corresponding clinical information. The GEPIA2 database is a comprehensive online tool that allows for the analysis of gene expression profiles across various cancer types, including LUAD [32]. It leverages data from TCGA and GTEx to provide insights into the mRNA expression levels of genes in different cancer stages, clinical subgroups, and tissue types. GEPIA2 offers an intuitive platform for researchers to assess gene expression and explore correlations with clinical features, providing a valuable resource for the identification of potential biomarkers and therapeutic targets. In our study, GEPIA2 was employed to investigate the expression of PTTG family genes (across different stages of LUAD. Furthermore, the GEO database, a vast repository of publicly available gene expression data, played a crucial role in validating and corroborating our findings in LUAD patients [33]. GEO ensures sample quality and minimizes biases through rigorous data curation and annotation processes. It requires researchers to adhere to standardized protocols for data submission, including detailed descriptions of sample characteristics and experimental procedures. This transparency allows users to assess the reliability of the data and consider potential biases during analysis. We acquired the standardized matrix profile (*series matrix.txt) of GSE32863, a microarray dataset consisting of expression profiles of 58 LUAD and 58 control tissue samples. Later on, the “limma” (linear models for microarray data) R package was used to discern the expression levels of PTTG family genes between LUAD and control samples, applying criteria of False Discovery Rate (FDR) < 0.05 and an absolute log2fold change (FC) > 1.

### Promoter methylation level validation of the PTTG family genes across additional datasets

MEXPRESS database, an invaluable resource in our study, provided a dynamic platform for exploring and visualizing DNA methylation and gene expression data [34]. With its user-friendly interface, MEXPRESS facilitated a comprehensive analysis of the intricate relationship between DNA methylation and gene expression patterns. In the present study, this database was utilized to validate the promoter methylation level of the PTTG family genes in the context of LUAD.

### Mutational landscape of PTTGP family genes

cBioPortal database [35] emerged as a cornerstone in our study, providing a robust platform for exploring the mutational landscape of PTTG family genes in LUAD patients. The interactive features of cBioPortal enhanced our ability to dissect complex molecular landscapes, fostering a deeper understanding of genetic mutations in PTTG genes across LUAD patients.

### Prognostic model development

The KM plotter tool [36] significantly contributed to our study by offering a powerful resource for survival analysis based on gene expression data. Leveraging Kaplan-Meier survival curves, this tool allowed us to assess the impact of PTTG gene expression on patient survival across LUAD patients.

The construction of the prediction model involved utilizing the Lasso and multivariate Cox proportional hazard regression analysis. The “survival” package in the R language [37] facilitated this process. TCGA-LUAD dataset serve as the training dataset while the “GSE72094, GSE68465, GSE63459, GSE50081, GSE42127, GSE41271, GSE37745, GSE31546, GSE3141, GSE31210, GSE30219, GSE29016, GSE29013, GSE26939, GSE19188, GSE14814, GSE13213, and GSE11969 datasets” were employed for validation. Positive coefficients in the analysis indicated an increased risk of an event (e.g., death), while negative coefficients suggested reduced risk, with their magnitudes reflecting the impact of variables on hazard rates. This information was instrumental in building prognostic models for survival outcomes. The formula for the prognostic model in LUAD patients was expressed as follows: risk score = the sum of the multivariate Cox regression coefficient variation of each mRNA.

### Correlation of PTTG family genes with immune modulators

The TISIDB [38] is a comprehensive online resource designed to provide insights into the interactions between tumors and the immune system. It integrates multiple types of data, including gene expression profiles, immune cell infiltration, and immune-related gene expressions across various cancer types. TISIDB allows users to explore tumor-immune interactions, identify immune biomarkers, and analyze immune cell compositions in the tumor microenvironment. TISIDB was used in this study to evaluate correlations between PTTG family genes and immune modulators in LUAD.

### Gene enrichment analysis

The DAVID tool [39] played a pivotal role in our study, facilitating comprehensive gene enrichment analysis of the PTTG family genes. Its robust functionality allowed us to uncover enriched biological themes, pathways, and molecular functions associated with the analyzed genes.

### Exploration of PTTG expression regulatory drugs

In our research, the DrugBank database [40] served as an indispensable resource, offering a comprehensive platform for the exploration of drugs related to PTTG family genes. This database facilitated the identification and evaluation of potential drugs capable of regulating the expression of PTTG family genes.

### SiRNA transfection for PTTG gene knockdown

siRNAs targeting PTTG1 and PTTG2 were purchased from Abcam (Cambridge, UK). To achieve silencing of the PTTG family genes, A549 and H1975 LUAD cell lines were transfected with the respective siRNA using INTERFERin transfection reagent (INTERFERin, France). The cells were cultured in appropriate media and transfected according to the manufacturer’s protocol to ensure efficient knockdown of the target genes. Western Blot analysis was performed according to protocols previously published in the literature [41, 42].

### Cell proliferation assay

Cell proliferation was assessed using the CCK-8 assay (Abcam, UK). A549 and H1975 LUAD cell lines were seeded in 96-well plates at a density of 1 × 10⁴ cells per well. After 24, 48, and 72 h of incubation, 10 µL of CCK-8 solution was added to each well, and the absorbance was measured at 450 nm using a microplate reader to quantify cell viability.

### Colony formation assay

For colony formation, A549 and H1975 cells were seeded in 6-well plates at a density of 500 cells per well. After 10–14 days of culture, colonies were fixed with 4% paraformaldehyde and stained with crystal violet. Colonies consisting of at least 50 cells were counted under a microscope.

### Wound healing assay

Wound healing assays were performed by creating a scratch in a confluent monolayer of A549 and H1975 cells using a sterile pipette tip. Cells were treated with siRNA and allowed to migrate into the wound area for 24 h. The wound closure was observed and photographed at 0 and 24 h. The wound healing percentage was calculated by measuring the wound area using ImageJ software.

### Molecular docking analysis

To evaluate the binding affinities between Calcitriol and PTTG1/2 proteins, molecular docking was performed using the CB-DOCK web server [43]. The SDF structure of Calcitriol (PubChem ID: 5280453) was sourced from PubChem, while PTTG1/2 structures were generated via SwissModel with 98% and 99% similarity, respectively. Docking calculations were done to determine binding energies, and the most favorable conformation based on hydrogen bond energy was visualized using PyMOL (version 2.5.2).

### Statistical analysis

To enhance the reliability of in silico findings, multiple hypothesis testing corrections were applied to relevant bioinformatic analyses. Specifically, the Benjamini-Hochberg false discovery rate (FDR) method was used to adjust *p*-values in differential gene expression analyses (limma), gene enrichment analysis (DAVID), and immune correlation studies (TISIDB). Data were analyzed using GraphPad Prism 8 and R software. For comparisons between two groups, a student’s t-test was used, while one-way ANOVA followed by Tukey’s post hoc test was applied for multiple group comparisons. Statistical significance was set at p* < 0.05. p** < 0.01, and p*** < 0.001. Data are presented as means ± standard deviation (SD) from at least three independent experiments.

## Results

### RT-qPCR analysis of PTTG genes

**Fig. 1:**
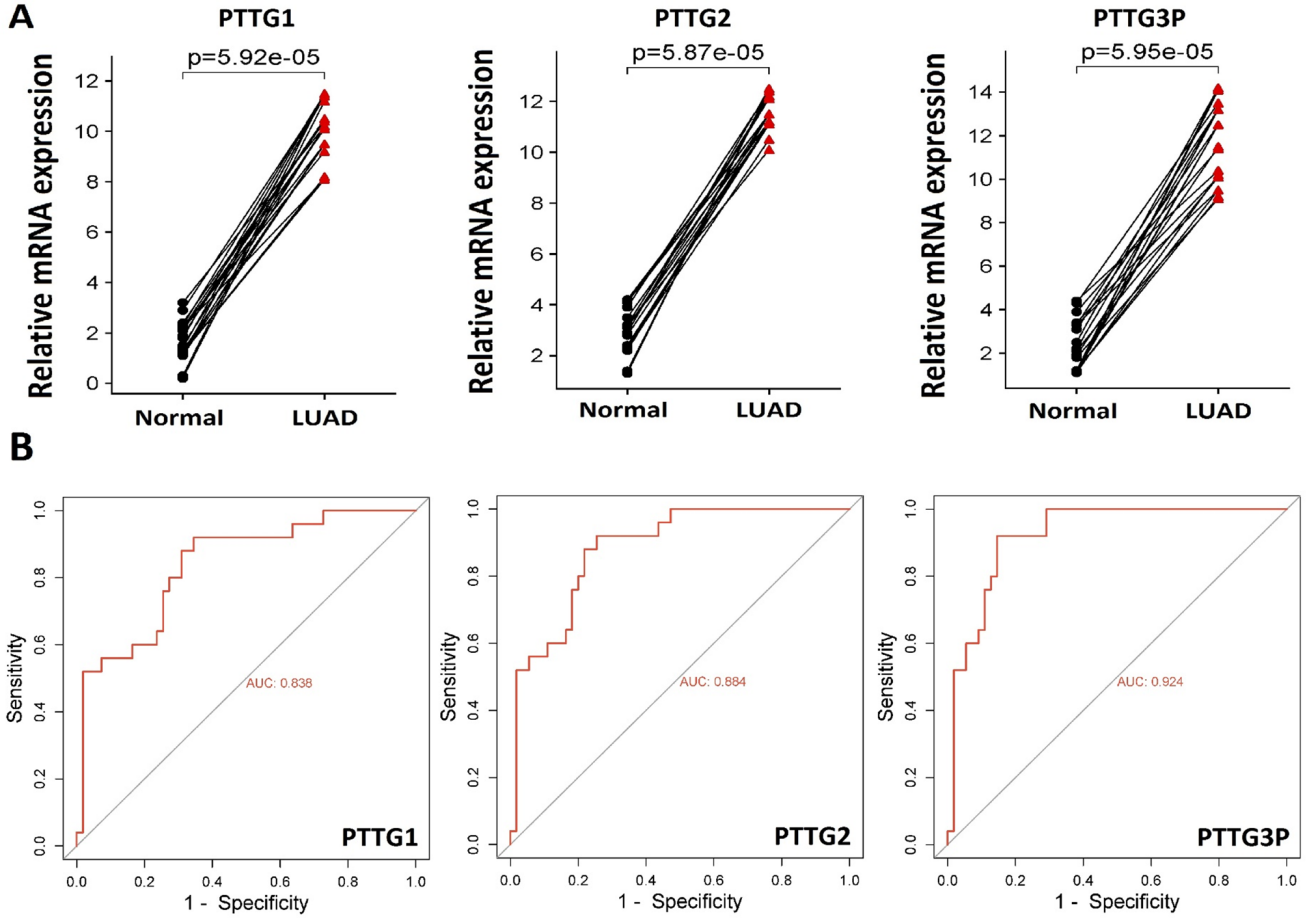
Illustrates the expression profiling and ROC analysis of the PTTG genes. (**A**) Relative expression of the PTTG genes in lung adenocarcinoma (LUAD) and adjacent control cell line groups using RT-qPCR, and (**B**) Expression-based ROC analysis of the PTTG genes. Statistical comparisons were performed, and *p*-values are reported (*p* < 0.05 was considered statistically significant

The mRNA expression levels of PTTG1, PTTG2, and PTTG3P genes were assessed using RT-qPCR in 10 LUAD and 10 normal control cell lines. The results revealed a statistically significant increase (*p*-value < 0.05) in the mRNA expression of PTTG1, PTTG2, and PTTG3P in the LUAD cell lines group compared to the normal cell lines group (Fig. 1A). Furthermore, using this expression data, we analyzed the diagnostic potential of PTTG1, PTTG2, and PTTG3P in distinguishing between LUAD patients and normal individuals, employing the creation of ROC curves. Expression-based ROC curves demonstrated that the elevated expression of PTTG1, PTTG2, and PTTG3P genes effectively distinguishes between LUAD patients and normal individuals (Fig. 1B).

### Bisulfite sequencing-based promoter methylation analysis

**Fig. 2:**
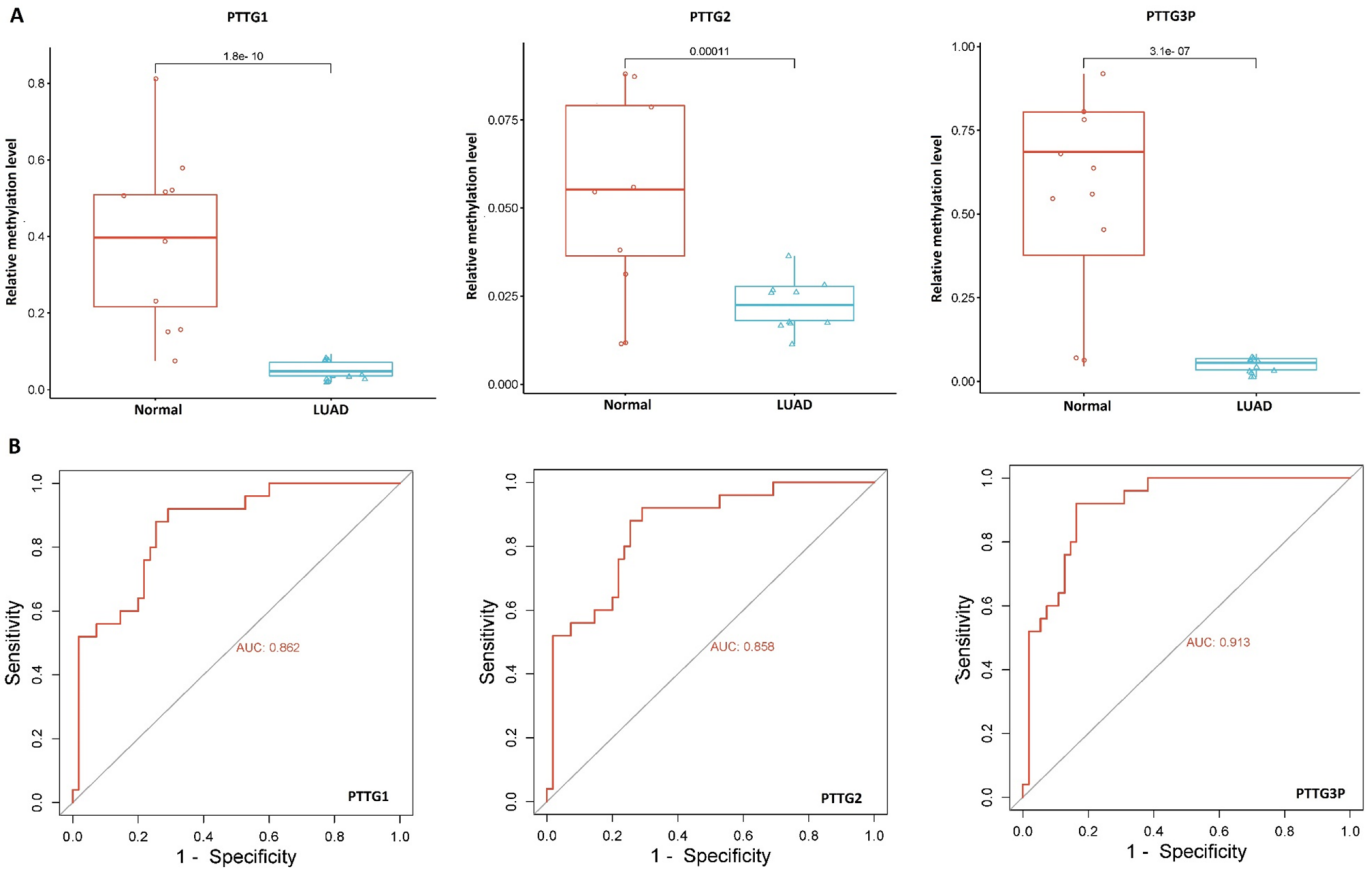
Displays the profiling of promoter methylation levels and ROC analysis of the PTTG gene using bisulfite sequencing. (**A**) Relative promoter methylation levels of the PTTG gene in lung adenocarcinoma (LUAD) and normal control cell line groups assessed through bisulfite sequencing, and (**B**) ROC analysis based on promoter methylation levels of the PTTG genes. Statistical comparisons were performed, and *p*-values are reported (*p* < 0.05 was considered statistically significant

Previous investigations have emphasized the significance of DNA promoter methylation patterns in influencing tumorigenesis [44, 45]. In our pursuit to uncover the relationship between the expression of PTTG1, PTTG2, and PTTG3P and DNA methylation, we assessed the methylation levels of these genes in 10 LUAD cell lines and 10 normal control cell lines using bisulfite sequencing analysis. Figure 2A illustrates methylation plots for PTTG1 (CpG dinucleotides 160420900–160429700), PTTG2 (CpG dinucleotides 37959500–37962100), and PTTG3P (CpG dinucleotides 44848000–44876000) in LUAD and control cell line samples. The findings revealed significant (*p*-value < 0.05) hypomethylation of PTTG1, PTTG2, and PTTG3P in LUAD cell line samples compared to normal control cell lines (Fig. 2A), suggesting that DNA methylation might contribute to the elevated expression of PTTG1, PTTG2, and PTTG3P. Additionally, the ROC curves based on promoter methylation illustrated that the hypomethylation patterns of PTTG1, PTTG2, and PTTG3P genes are also highly effective in discerning between individuals with LUAD and those without the condition (Fig. 2B).

### Expression validation of the PTTG family genes

**Fig. 3:**
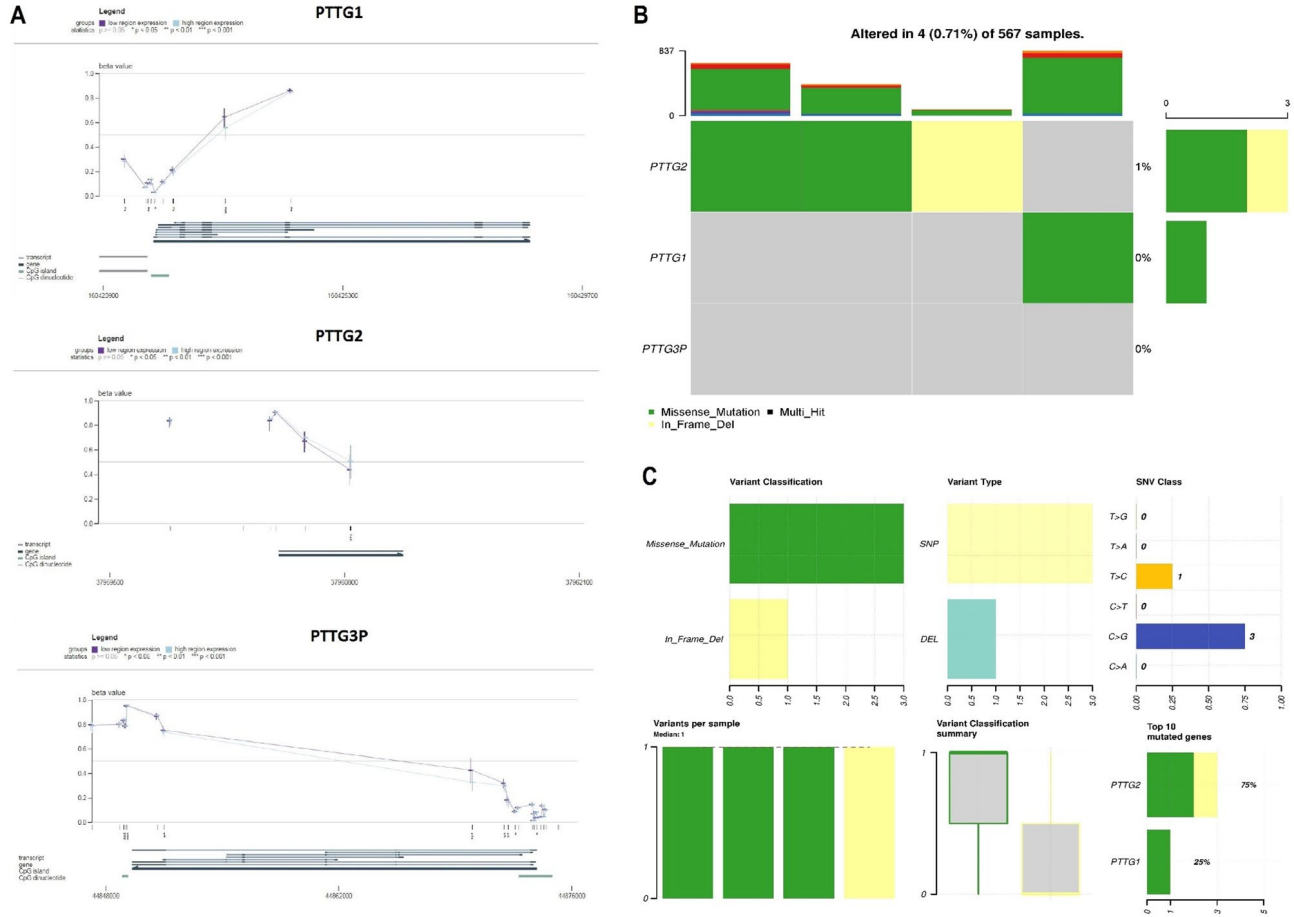
Displays the analysis of promoter methylation and mutations in the PTTG genes across TCGA lung adenocarcinoma (LUAD) and normal control samples using the MEXPRESS and cBioPortal databases. (**A**) Promoter methylation levels of PTTG family genes across LUAD samples. (**B**) Percentage of mutated LUAD samples, and (**C**) Summary of the observed genetic alterations in PTTG genes across LUAD samples. Group comparisons were performed using statistical tests, and *p*-values are reported; values with *p* < 0.05 were considered statistically significant

**Fig. 4:**
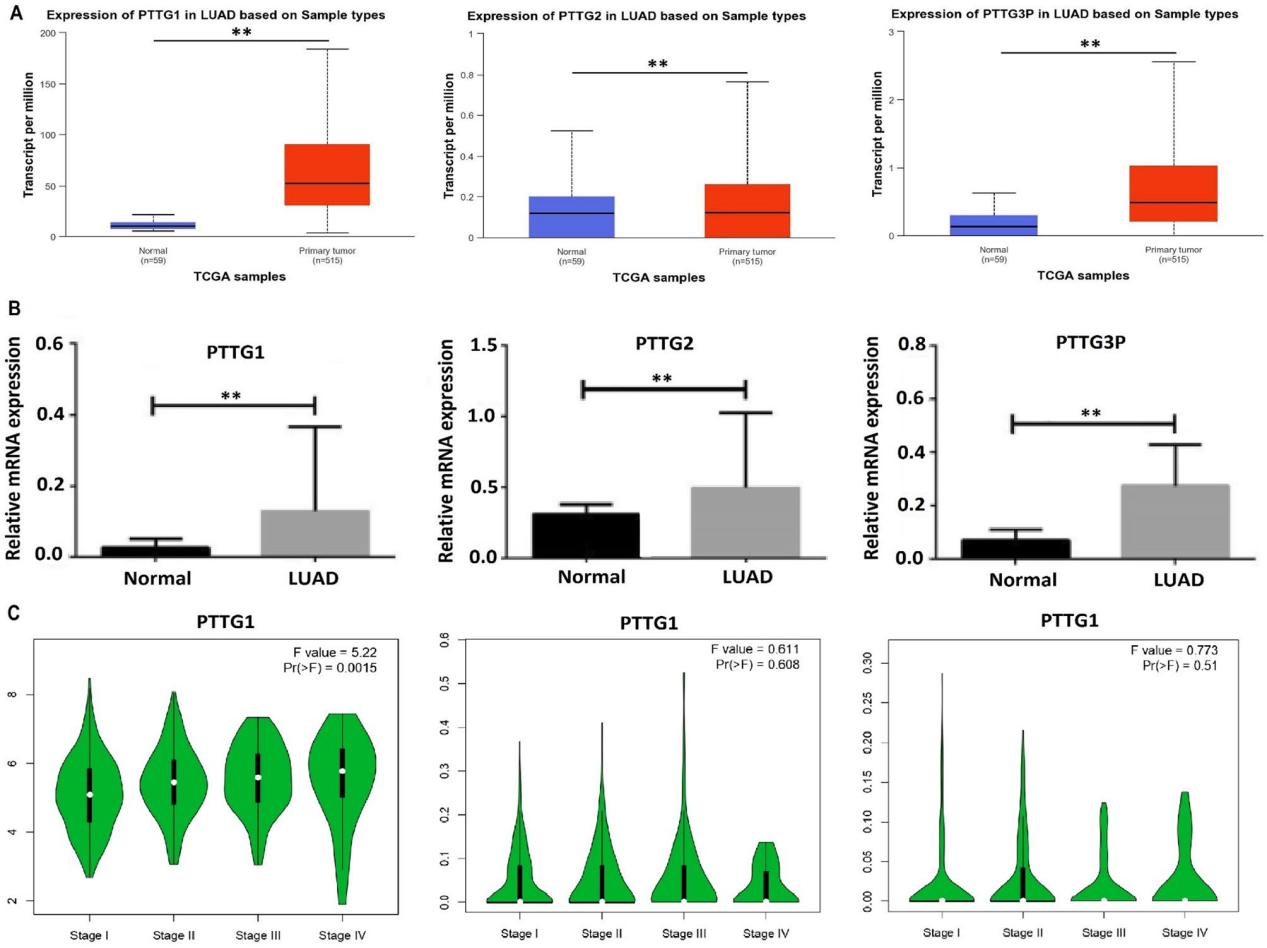
Illustrates the mRNA expression profiling of the PTTG genes across TCGA and GEO datasets. (**A**) Box plot representation of PTTG gene mRNA expression in the TCGA lung adenocarcinoma (LUAD) sample group and normal control group, and (**B**) Box plot representation of PTTG gene mRNA expression in the GEO LUAD sample group and normal control group. (**C**) Box plot representation of PTTG gene mRNA expression across different LUAD stages. Group differences were evaluated using statistical tests, and adjusted *p*-values (Benjamini–Hochberg) are reported to account for multiple gene comparisons. Values with adjusted *p* < 0.05 were considered statistically significant

Upon validating the mRNA expression levels of PTTG1, PTTG2, and PTTG3P genes in both TCGA LUAD and GSE32863 datasets, the box plots illustrated a significant (*p*-value < 0.05) up-regulation of PTTG1, PTTG2, and PTTG3P in LUAD tissue samples compared to adjacent normal tissues (Fig. 3A-B). This reinforces the confirmation that PTTG1, PTTG2, and PTTG3P genes exhibit up-regulation in patients with LUAD. Moreover, in Fig. 4C, the gene expression across different stages of LUAD progression was assessed via the GEPIA2 database. For PTTG1, there was a significant increase in expression from Stage I to Stage IV (F value = 5.22, *p* = 0.0015), suggesting that PTTG1 expression may correlate with cancer stage progression (Fig. 4C). However, for PTTG2 and PTTG3P, there are no significant changes observed across the stages (F values = 0.611 and 0.773, respectively; *p* = 0.608 and 0.51), indicating that these genes might not have a strong association with cancer stage progression in LUAD (Fig. 4C).

### Promoter methylation level validation of the PTTG family genes

We proceeded to verify the promoter methylation levels of PTTG1, PTTG2, and PTTG3P genes in TCGA LUAD patients utilizing the MEXPRESS database. Our analysis revealed that tumors showcasing elevated expression of PTTG1, PTTG2, and PTTG3P genes exhibited significant (*p*-value < 0.05) reduced DNA methylation levels in LUAD samples compared to corresponding controls (Fig. 3A). These results imply that DNA methylation might contribute to the overexpression of PTTG1, PTTG2, and PTTG3P in LUAD.

### Exploring the mutational landscape of PTTGP family genes

The OncoPrint representation of PTTG1, PTTG2, and PTTG3P genes in the cBioPortal database served to display mutations using data derived from 587 LUAD patients in the TCGA. Among these patients, only 4% exhibited mutations in the PTTG1 and PTTG2 genes, while no mutations were observed in the PTTG3P genes. Notably, the highest mutation rate was associated with PTTG2 (1%), wherein missense mutation (C > G) emerged as the most prevalent mutation type (Fig. 3B-C).

### Survival analysis and the development of the prognostic model based on the PTTG genes

**Fig. 5:**
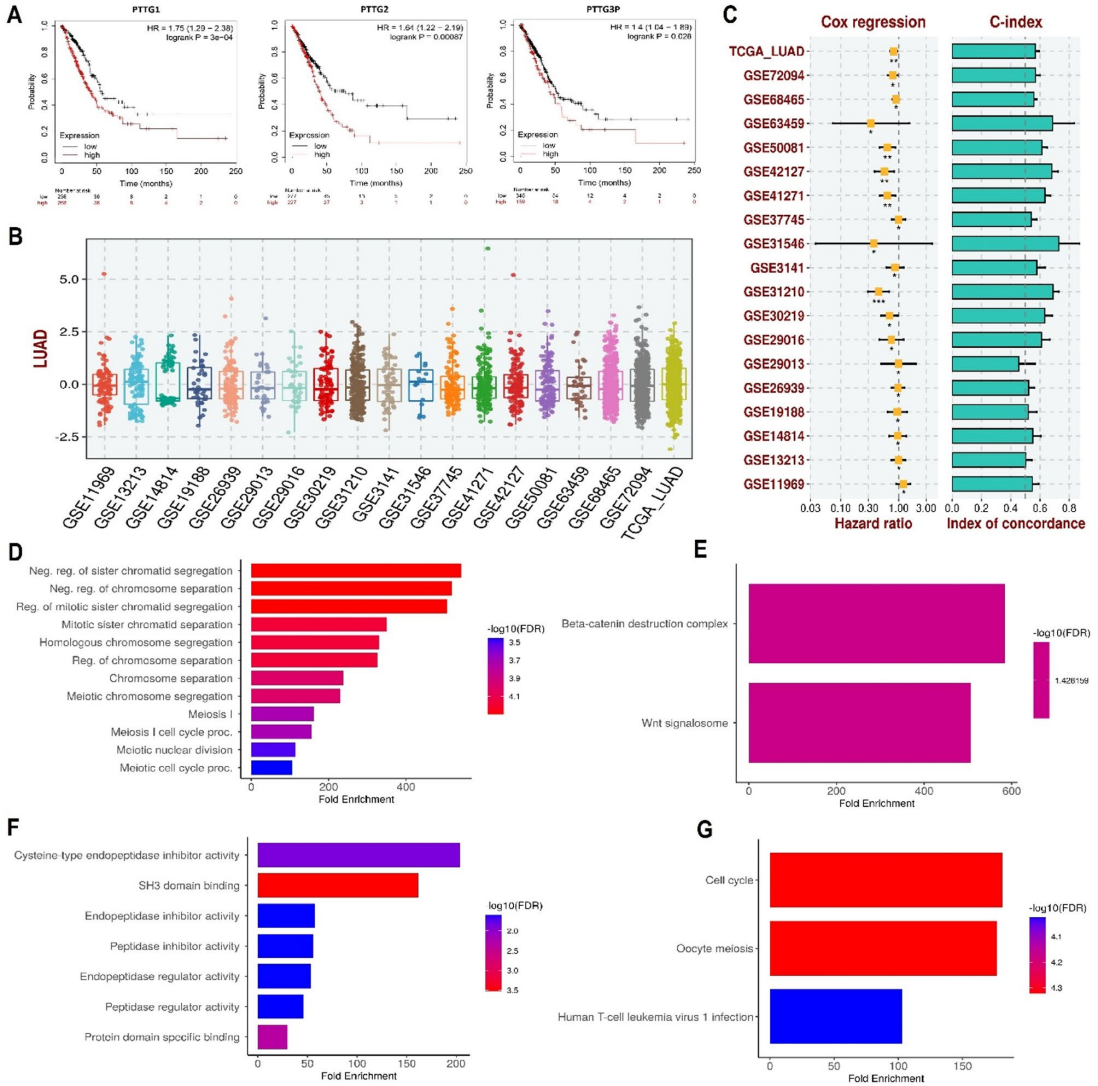
Depicts the survival analysis, development of the prognostic model, and gene enrichment analysis of PTTG genes. (**A**) KM plotter-based survival analysis of the PTTG genes in TCGA lung adenocarcinoma (LUAD) samples, (**B**) Univariate Cox regression analysis, and (**C**) Risk scores. (**D**) PTTG genes-associated CC terms, (**E**) PTTG genes-associated BP terms, (**F**) PTTG genes-associated MF terms, and (**G**) PTTG genes-associated KEGG terms. Group comparisons were performed using statistical tests, and *p*-values are reported; values with *p* < 0.05 were considered statistically significant

Using expression data and clinical details from LUAD samples in the KM plotter, we investigated the links between the expression levels of PTTG1, PTTG2, and PTTG3P genes and LUAD patient survival. The outcomes of the KM plotter analysis revealed that the elevated expression of PTTG1, PTTG2, and PTTG3P genes correlated with the poorest prognosis among LUAD patients (Fig. 5A). However, factors such as treatment regimens and comorbidities can significantly impact patient prognosis. For example, patients receiving different therapeutic interventions, such as surgery, chemotherapy, or targeted therapy, may have varying survival outcomes. Additionally, the presence of comorbidities, such as cardiovascular disease or diabetes, could influence patient response to treatment and overall survival.

Therefore, we developed a prognostic model utilizing PTTG1, PTTG2, and PTTG3P genes, with the TCGA_LUAD dataset serving as the training dataset. The TCGA_LUAD dataset was chosen as the training dataset based on its larger sample size, which increases statistical power and enables the capture of diverse characteristics within the target population. The “GSE72094, GSE68465, GSE63459, GSE50081, GSE42127, GSE41271, GSE37745, GSE31546, GSE3141, GSE31210, GSE30219, GSE29016, GSE29013, GSE26939, GSE19188, GSE14814, GSE13213, and GSE11969” datasets functioned as validation datasets. The validation datasets also encompass the qualities of the training dataset but with an emphasis on their independence from the training dataset to avoid overfitting. These datasets represented a diverse set of patients similar to those in the training dataset but sourced from different cohorts or studies to assess the model’s performance across various populations. Employing a stepwise Cox regression model integrating hazard ratio, c-index, and risk score parameters, we constructed the prognostic model. Evaluation of our predictive prognostic model across all analyzed datasets using the c-index revealed its effective and robust capacity to assess the prognosis of LUAD patients (Fig. 5B-C).

### Gene enrichment analysis

We analyzed PTTG1, PTTG2, and PTTG3P genes to figure out their GO and KEGG pathways in LUAD. In the CC, “Negative regulation of sister chromatid segregation, Negative regulation of chromosome separation, and Reg. of mitotic sister chromatid segregation” etc., terms were significantly associated with the PTTG1, PTTG2, and PTTG3P (Fig. 5D). Concerning MF, the “Beta-catenin destruction complex and Wnt signalosome” terms were closely associated with the PTTG1, PTTG2, and PTTG3P (Fig. 5E). In BP, some vital functions including “Cysteine-type endopeptidase inhibitor activity, SH3 domain binding, and endopeptidase inhibitor activity” etc., terms were significantly associated with the PTTG1, PTTG2, and PTTG3P (Fig. 5F). Given the significant associations observed between PTTG1, PTTG2, and PTTG3P and various cellular processes, including sister chromatid segregation, chromosome separation, and regulation of mitotic processes, it’s evident that the up-regulation these genes play multifaceted roles in LUAD pathogenesis. The identified associations with beta-catenin destruction complex and Wnt signalosome highlight potential involvement of up-regulated PTTG in crucial signaling pathways implicated in LUAD progression. Moreover, the enrichment of terms related to endopeptidase inhibitor activity and SH3 domain binding suggests potential roles in modulating protein-protein interactions and protease activity, which are known to influence tumor growth and metastasis. Moreover, PTTG1, PTTG2, and PTTG3P-enriched KEGG pathways include “Cell cycle, Oocyte meiosis, and Human T-cell leukemia virus 1 infection” etc., (Fig. 5G). The enrichment of the “Cell cycle” pathway implies that PTTG genes may disrupt normal cell division regulation, contributing to uncontrolled tumor growth in LUAD. Additionally, the presence of the “Oocyte meiosis” pathway enrichment suggests broader roles for PTTG genes beyond cancer pathways, possibly involving regulatory mechanisms conserved across different cell types. Furthermore, the enrichment of the “Human T-cell leukemia virus 1 infection” pathway hints at potential interactions between PTTG genes and viral pathways in LUAD, raising intriguing possibilities for further investigation into viral-induced cellular changes in cancer development.

### Correlation of PTTG family genes with immune modulators

**Fig. 6:**
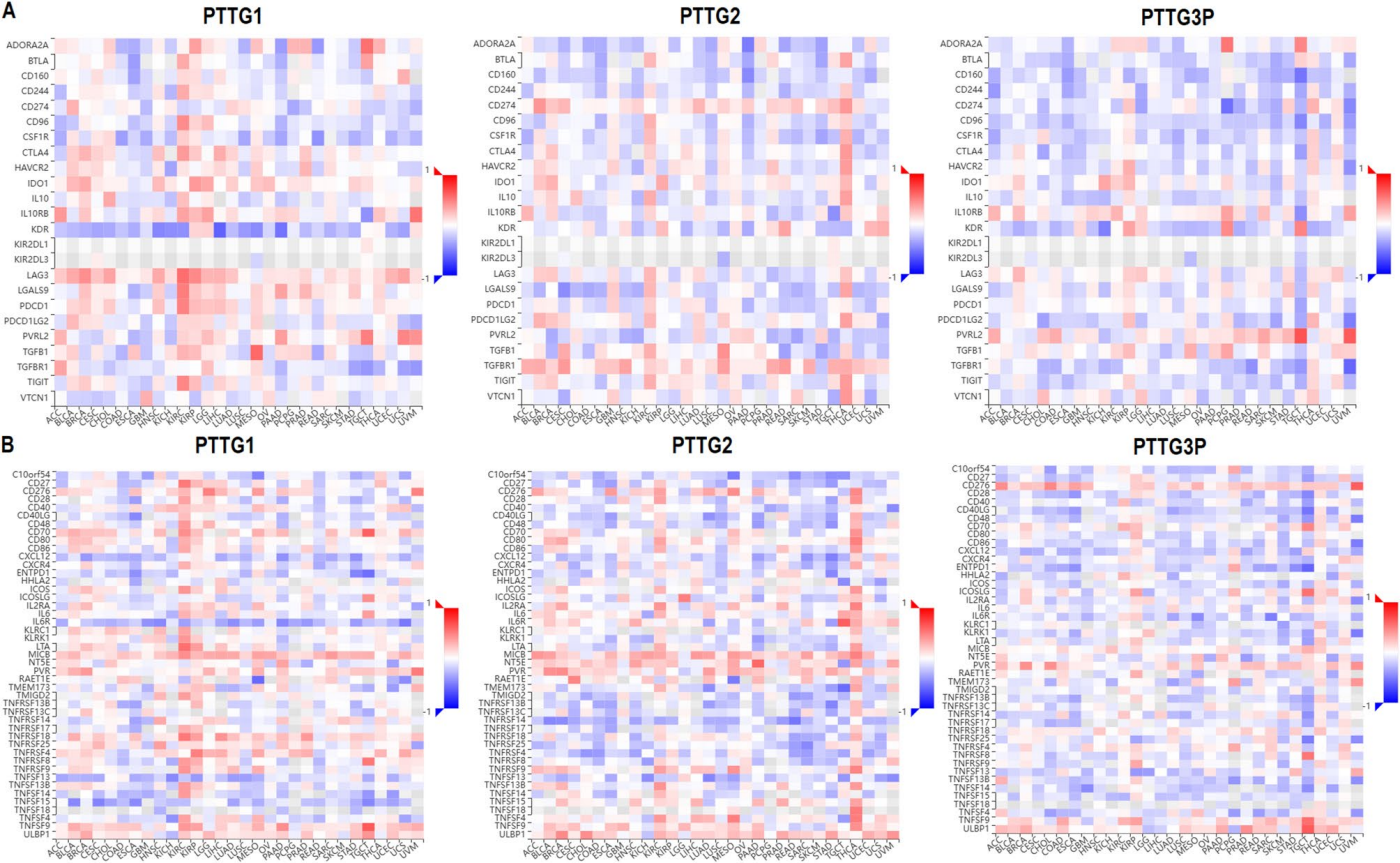
shows correlation analysis of PTTG1, PTTG2, and PTTG3P gene expression with immune-related genes in LUAD using the TISIDB database. (**A**) Positive correlations (highlighted in red) between PTTG genes and immune inhibitor genes (**B**) Negative correlations (depicted in blue) between PTTG genes and immune stimulator genes. Group comparisons were performed using statistical tests, and *p*-values are reported; values with *p* < 0.05 were considered statistically significant

In this part of the study, the correlation between PTTG1, PTTG2, and PTTG3P gene expression and immune-related genes was analyzed using the TISIDB database. Figure 6A focused on the correlation with immune inhibitor genes, where positive correlations, highlighted in red, indicated that higher expression levels of PTTG genes corresponded with increased expression of immune inhibitor genes such as PDCD1, TIGIT, and CTLA4 (Fig. 6A). This suggests that PTTG genes may contribute to immune suppression within the tumor microenvironment, promoting immune evasion. In Fig. 6B, the analysis of immune stimulator genes revealed negative correlations (depicted in blue), where elevated PTTG gene expression was associated with decreased expression of immune stimulators like CD27, ICOS, and CD40LG (Fig. 6B). This inverse relationship further supports the idea that PTTG genes may suppress immune activation, thereby facilitating immune escape in LUAD.

### PTTG1/2 knockdown and functional assays

**Fig. 7:**
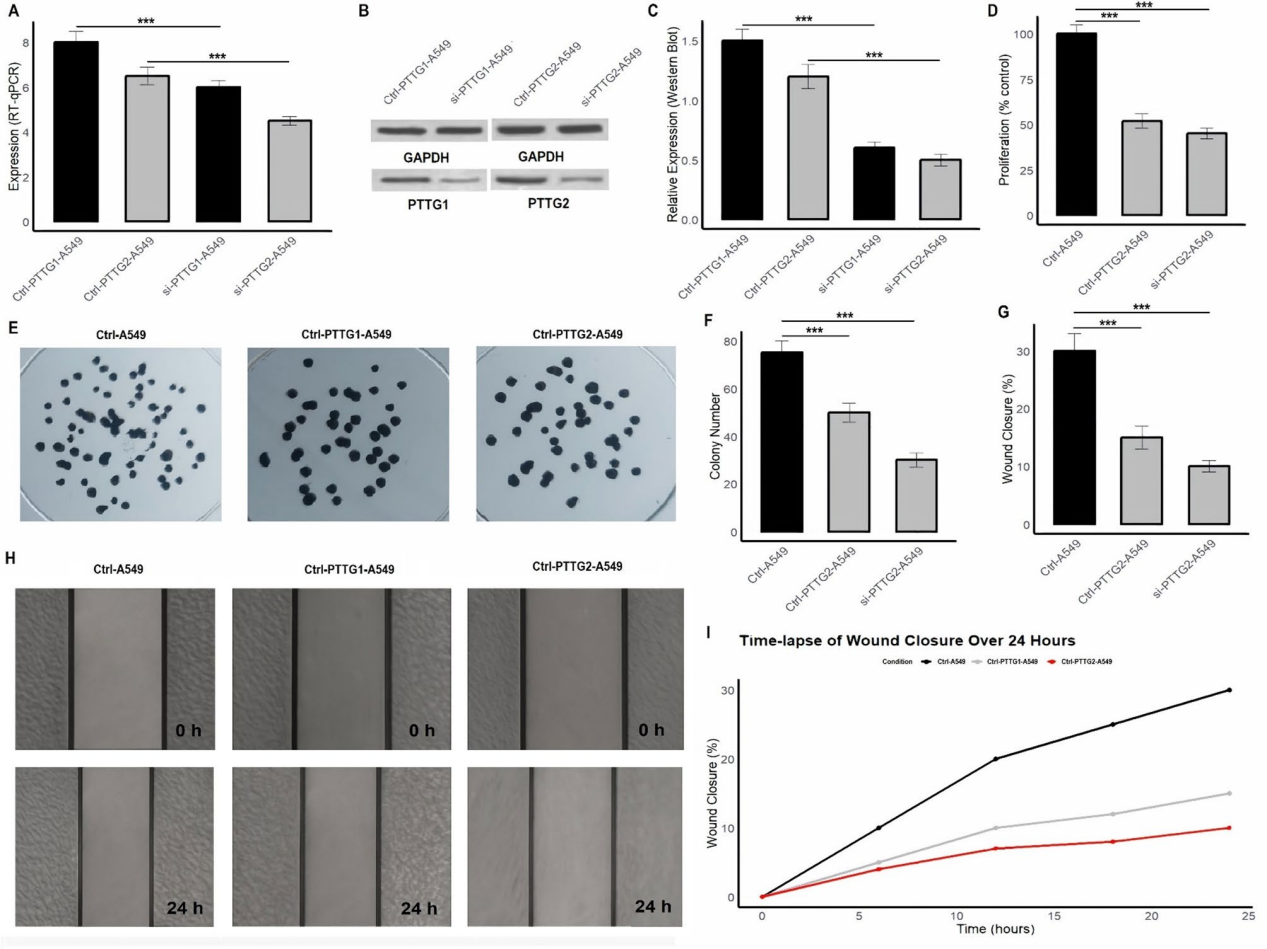
Show the effects of PTTG1 and PTTG2 knockdown in A549 cells are shown. (**A-C**) The RT-qPCR and Western Blot analyses confirm a significant reduction in the mRNA and protein expression levels of PTTG1 and PTTG2 after siRNA treatment. (**D**) Proliferation assays revealed a marked decrease in cell viability in the si-PTTG1 and si-PTTG2 treated groups compared to control cells, indicating that the knockdown of these genes impairs cell growth. (**E-F**) The colony formation assay demonstrated a reduction in the number of colonies formed by the silenced cells, further supporting the role of PTTG1 and PTTG2 in promoting cell growth and survival. (**G-I**) The wound healing assay showed significant delays in wound closure for si-PTTG1 and si-PTTG2 treated cells, suggesting that their knockdown impairs cell motility and migration. A p***-value < 0.001 was considered significant

**Fig. 8:**
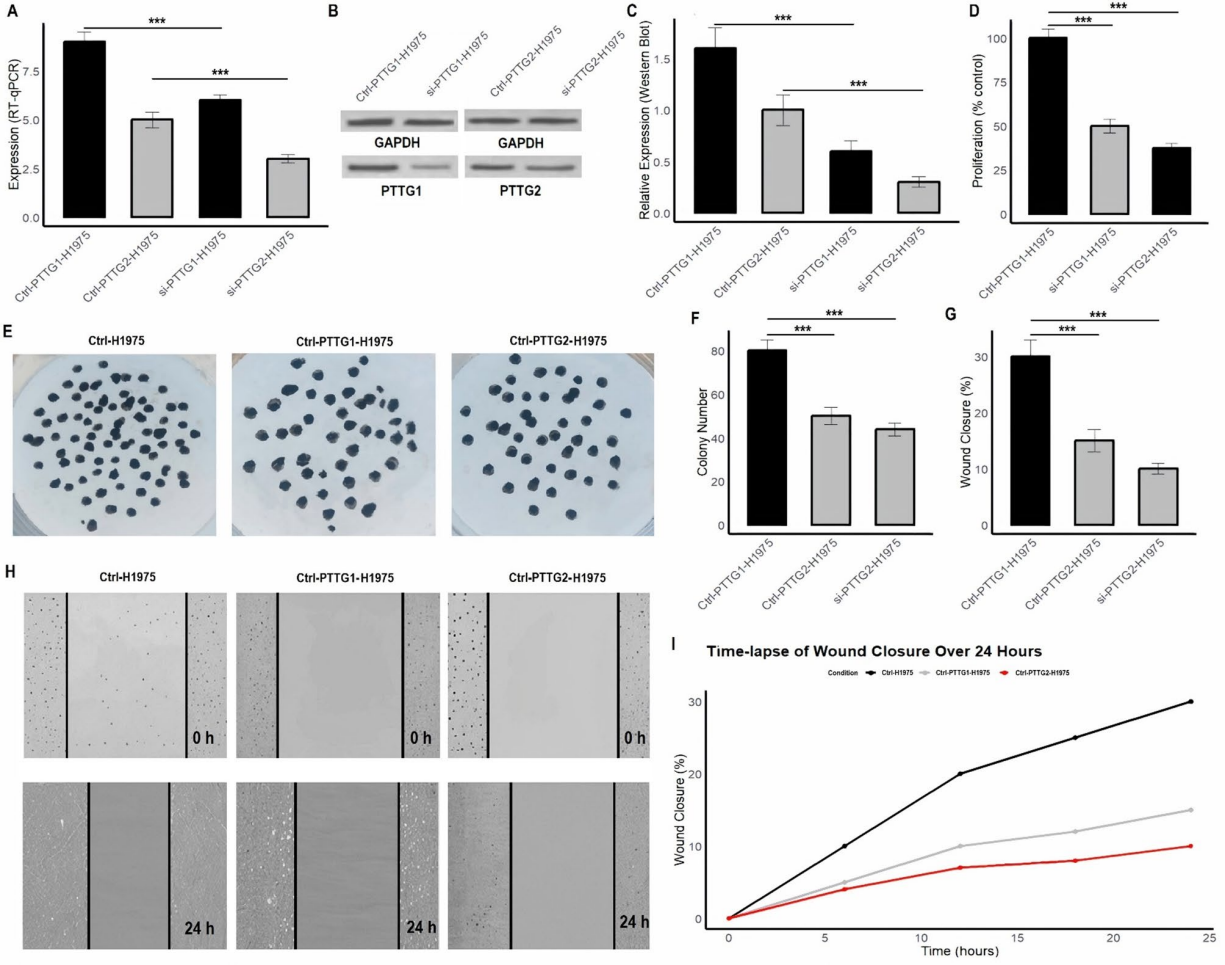
Depict the effects of PTTG1 and PTTG2 knockdown in H1975 cells are presented. (**A-C**) RT-qPCR and Western Blot results show a significant decrease in the mRNA and protein levels of PTTG1 and PTTG2 following siRNA treatment. (**D**) Proliferation assays reveal a substantial reduction in cell viability in the knockdown groups (si-PTTG1 and si-PTTG2) compared to control cells, highlighting the essential role of these genes in cell growth. (**E-F**) The colony formation assay indicates fewer colonies formed by the silenced cells, further confirming the involvement of PTTG1 and PTTG2 in cell survival and growth. (**G-I**) In the wound healing assay, the si-PTTG1 and si-PTTG2 treated H1975 cells exhibited significantly delayed wound closure, suggesting impaired migration and motility. These effects were more pronounced in H1975 cells compared to A549 cells, suggesting that the knockdown of PTTG1 and PTTG2 has a stronger impact on cell migration in H1975. A p***-value < 0.001 was considered significant

In this part of the study, the effects of PTTG1 and PTTG2 knockdown on the A549 and H1975 LUAD cell lines were investigated to assess their role in cell proliferation, colony formation, migration, and gene expression. PTTG1 and PTTG2 were silenced using siRNA in both cell lines, and various assays were conducted to evaluate the consequences of their knockdown. The results demonstrate a significant reduction in the mRNA and protein expression levels of PTTG1 and PTTG2 in both cell lines following siRNA treatment (Figs. 7A-C and 8A-C, and Supplementary data Fig. 1). Proliferation assays revealed a notable decrease in cell viability after knockdown, with A549 and H1975 cells showing reduced growth compared to control groups (Figs. 7D and 8D). Similarly, the colony formation assay indicated a reduction in the number of colonies formed by the silenced cells, further confirming the role of PTTG1 and PTTG2 in cell growth (Figs. 7E-F and 8E-F). In terms of cell migration, the wound healing assay demonstrated that knockdown of PTTG1 and PTTG2 led to a significant delay in wound closure, suggesting impaired cell motility (Figs. 7G-I and 8G-I). These effects were evident in both A549 and H1975 cells, but the knockdown had a more pronounced impact on the H1975 cells.

### Drug prediction and molecular docking analysis

**Fig. 9:**
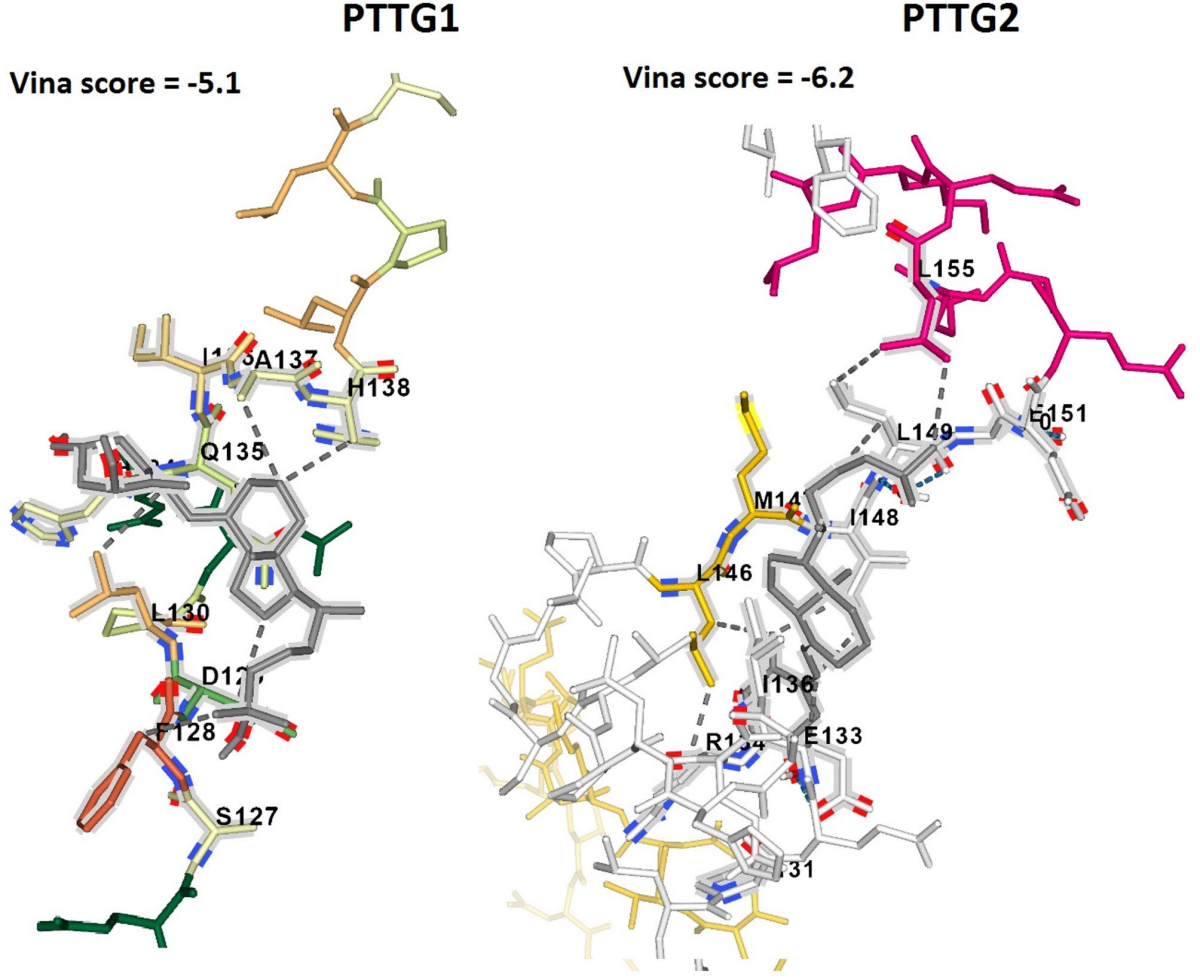
displays the molecular docking results of Calcitriol with PTTG1 and PTTG2 genes. The structures of PTTG1 and PTTG2 proteins are depicted in blue, while the Calcitriol drug is represented by gray molecules, illustrating their docking interactions with the target proteins

As mentioned earlier, PTTG3P is categorized as a pseudogene and does not possess the capability to encode a protein. Therefore, the DrugBank database was employed to explore potential drugs with the capacity to diminish the expression of PTTG1 and PTTG2 proteins in the context of LUAD treatment. Within this repository, Calcitriol was identified as a potential candidate predicted to interact with and possibly modulate the expression of both PTTG1 and PTTG2 genes, warranting further experimental investigation. To validate the impact of Calcitriol on expression reduction, molecular docking analysis was conducted. Docking results unveiled varying binding affinities of Calcitriol with PTTG1 and PTTG2, spanning from − 5.1 to -6.2 kcal/mol (Fig. 9). These binding affinities, falling within the range of -5.1 to -6.2 kcal/mol, signify a relatively robust interaction between Calcitriol and the PTTG1 and PTTG2 proteins (Fig. 9). While molecular docking analysis provides valuable insights into potential drug-target interactions, it is essential to consider the reproducibility of these results and acknowledge potential limitations associated with in silico predictions. One critical aspect to address is the reliability of scoring functions used in docking studies, as different scoring algorithms may yield varying results and may not always accurately reflect true binding energies. Additionally, structural inaccuracies in protein models or ligand conformations could introduce uncertainties in docking predictions. Therefore, it is crucial to validate docking results using experimental methods such as binding assays or structural biology techniques.

## Discussion

As the most prevalent form of lung cancer [46, 47], significant advancements in the diagnosis, prognosis, and treatment of LUAD are crucial. The PTTG family, encompassing PTTG1, PTTG2, and PTTG3P, represents a newly recognized gene class. Among these three homologous genes, PTTG1 has undergone extensive investigation and has been established as closely linked to the onset and progression of various human cancer types, including pituitary tumors [48], gliomas [16], breast [4, 49], thyroid [50], bladder [17, 51], ovarian [9], and prostate cancers [18]. Moreover, another investigation has highlighted the substantial up-regulation of PTTG1 in non-small cell lung cancer (NSCLC), underscoring its involvement in the initiation and advancement of NSCLC. Nevertheless, a comprehensive analysis addressing the expression and prognostic significance of PTTG1 in LUAD has not been undertaken to date. Additionally, while PTTG2 and PTTG3P have been linked to tumor development [11, 13], no prior study, to the best of our knowledge, has delved into their expression patterns and roles in LUAD. Thus, the current study systematically explores the expression and prognostic implications of the PTTG family genes in LUAD.

The current study conducted a thorough examination of the mRNA expression of PTTG family genes in clinical LUAD cell lines and tissue. The findings revealed a notable up-regulation of PTTG1 in cancerous tissues and cell lines when compared to normal counterparts, aligning with observations from previous studies in various cancer types [16–18]. However, consideration of factors such as tumor purity and stromal content is essential for the interpretation of gene expression data from bulk tumor samples. Therefore, incorporating computational deconvolution methods and histological assessments can provide insights into the influence of the tumor microenvironment on PTTG expression in LUAD.

EGFR and KRAS are well-established oncogenes implicated in LUAD pathogenesis, influencing various cellular processes such as proliferation, survival, and metastasis [52]. PTTG genes, known for their involvement in cell cycle regulation, may synergize with EGFR and KRAS signaling pathways to promote tumor growth and aggressiveness. Co-activation of PTTG genes and EGFR/KRAS pathways could lead to enhanced downstream signaling cascades, resulting in increased cell proliferation, survival, and resistance to apoptosis. Alternatively, antagonistic interactions between PTTG genes and EGFR/KRAS pathways may exist, where one pathway suppresses the activity of the other. Dysregulation of PTTG genes may inhibit cell cycle progression and counteract the proliferative effects of EGFR/KRAS signaling, leading to reduced tumor growth. Conversely, activation of EGFR/KRAS pathways may suppress PTTG gene expression, thereby modulating cell cycle dynamics and limiting tumor progression. Understanding the precise nature of these interactions is critical for tailoring effective therapeutic strategies.

The Kaplan-Meier Plotter database was employed for a comprehensive evaluation of the prognostic implications of PTTG family genes in LUAD patients. The findings indicated that individuals with elevated expression of PTTG1, PTTG2, and PTTG3P in LUAD experienced a shortened overall survival compared to healthy controls. These results align with survival analyses from previous studies, reinforcing the role of PTTG family genes as prognostic markers across various cancer types [53, 54].

Additionally, an examination of promoter methylation and genetic alterations in PTTG1, PTTG2, and PTTG3P was conducted across samples from individuals with LUAD. The results unveiled hypomethylation and minimal mutations in these genes within LUAD patients, suggesting a correlation between promoter hypomethylation and the down-regulation of these genes in LUAD. The observed hypomethylation of PTTG genes in LUAD implies a potential mechanism underlying their down-regulation in cancer cells. This phenomenon highlights the epigenetic plasticity of PTTG gene expression profiles, suggesting that changes in DNA methylation status could dynamically regulate their expression levels over time. Understanding the dynamic nature of DNA methylation patterns and their impact on PTTG gene expression stability is essential for elucidating the regulatory mechanisms driving LUAD pathogenesis. It underscores the need for further investigation into the temporal dynamics of DNA methylation changes and their functional consequences on PTTG gene expression in LUAD.

Lastly, our investigation highlights Calcitriol as a valuable drug with potential applications in the treatment of LUAD in the context of PTTG family genes. Calcitriol, the active form of vitamin D, has been suggested as a potential therapeutic agent in cancer due to its diverse biological activities; however, further preclinical and clinical studies are needed to validate its efficacy [55]. Research indicates its potential to inhibit cancer cell proliferation, induce apoptosis, and suppress angiogenesis [55]. Furthermore, Calcitriol exhibits immune-modulatory effects, contributing to the body’s defense against cancer [56]. Ongoing clinical studies are exploring Calcitriol’s efficacy in various cancers, including breast, prostate, and colorectal cancers [57]. However, the pharmacokinetic properties of Calcitriol, such as rapid metabolism and short half-life, pose challenges for achieving sustained therapeutic levels. Moreover, the potential for off-target effects, including hypercalcemia and renal toxicity, necessitates careful dose titration and monitoring in clinical settings. Additionally, clinical translation faces hurdles related to formulation optimization to enhance bioavailability and minimize adverse effects. Strategies like nanoparticle-based delivery systems may help overcome these challenges. Clinical trials evaluating Calcitriol as an adjuvant or combination therapy in LUAD are underway, highlighting its potential as a therapeutic adjunct. Further research into optimizing dosing regimens, identifying predictive biomarkers, and elucidating synergistic interactions with existing treatments is crucial for realizing the therapeutic benefits of Calcitriol in LUAD management.

Our study has several limitations that should be acknowledged. First, although the sample size was sufficient for the conducted analyses, it may still limit the generalizability of the findings to broader LUAD populations. Second, the investigation was restricted to a selected subset of PTTG genes, leaving the wider landscape of genes involved in LUAD pathogenesis unexplored. Third, potential sources of bias exist, including dataset selection criteria and variations in data completeness across public platforms, which may affect the robustness of the results. Fourth, the lack of in vivo validation, such as studies in animal models or patient-derived xenografts (PDX), constrains the translational relevance of the therapeutic predictions. Fifth, molecular dynamics simulations of the docking complexes were not performed, which limits our ability to assess the stability of the predicted compound–target interactions. Finally, the proposed link between PTTG expression and key oncogenic drivers in LUAD (including EGFR and KRAS) remains hypothetical, as co-expression and pathway co-enrichment analyses have not yet been performed. Future studies addressing these limitations—through rigorous in vivo functional experiments, molecular dynamics simulations, and targeted co-expression/pathway analyses—will be essential to strengthen the mechanistic and translational significance of our findings.

## Conclusion

This study provides significant insights into the role of PTTG1, PTTG2, and PTTG3P in LUAD by investigating their expression profiles, methylation patterns, mutational landscape, and potential as therapeutic targets. RT-qPCR revealed a significant upregulation of PTTG1, PTTG2, and PTTG3P in LUAD cell lines and tissue samples compared to normal controls, suggesting that these genes may contribute to the malignant progression of LUAD. Our findings from the methylation analysis indicated that PTTG1, PTTG2, and PTTG3P exhibit hypomethylation in LUAD cells, which may underlie their increased expression, emphasizing the role of epigenetic modifications in cancer development. Additionally, molecular docking studies identified Calcitriol as a potential therapeutic agent capable of targeting PTTG1 and PTTG2, with strong binding affinities, though further experimental validation is required to confirm these findings. The immune correlation analysis using the TISIDB database revealed that the upregulation of PTTG genes is linked to immune evasion mechanisms, including increased expression of immune inhibitors (e.g., PDCD1, TIGIT, CTLA4) and reduced expression of immune stimulators (e.g., CD27, ICOS, CD40LG), suggesting that PTTG genes may play a role in modulating the tumor microenvironment by promoting immune suppression and facilitating immune escape in LUAD. In functional assays, the knockdown of PTTG1 and PTTG2 in A549 and H1975 LUAD cell lines led to significant reductions in proliferation, colony formation, and migration, highlighting the essential role of these genes in promoting cell growth and motility. These findings suggest that targeting PTTG1 and PTTG2 could serve as a therapeutic strategy for LUAD, potentially inhibiting tumor progression and metastasis. While this study provides compelling evidence for the role of PTTG genes in LUAD, several limitations should be considered. The in silico molecular docking predictions require experimental validation, and cell line models may not fully capture the complexity of in vivo LUAD. Future research involving in vivo models, clinical trials, and further validation of PTTG-targeting therapies will be essential to determine the clinical relevance and potential therapeutic benefits of targeting these genes in LUAD patients. Overall, this study highlights the significance of PTTG1 and PTTG2 in LUAD and presents a strong case for their potential as biomarkers and therapeutic targets, offering valuable directions for future cancer research and treatment development.

## Supplementary Information

Below is the link to the electronic supplementary material.

Supplementary Material 1

## Data Availability

Any type of data supporting the findings of this study are available from the corresponding author upon reasonable request.
