# Correction: Decoding the PTTG family’s contribution to LUAD pathogenesis: a comprehensive study on expression, epigenetics, and therapeutic interventions

**DOI:** 10.1186/s41065-025-00582-6

**Published:** 2025-10-15

**Authors:** Jinna Di, Li Tian, Fan Yan, Zhang Zhe, Cai Lin, Liu Jingyu

**Affiliations:** 1 Department of Respiratory and Critical Care Medicine, The Third Affiliated Hospital of Jinzhou Medical University, No. 2 Section 5 Heping Road, Linghe District, Jinzhou, 121000 Liaoning China; 2 Department of Respiratory and Critical Care Medicine, The First Affiliated Hospital of Jinzhou Medical University, No. 2 Section 5 Guta Road, Renmin District, Jinzhou, 121000 Liaoning China

**Correction: Hereditas 162**, **175 (2025)**

10.1186/s41065-025-00545-x

Following publication of the original article [1], the author reported that the first author was mistakenly captured as a corresponding author. Liu Jingyu should be the sole corresponding author.

The original article has been updated.
