# Supplementary figures and images for: Decoding the PTTG family’s contribution to LUAD pathogenesis: a comprehensive study on expression, epigenetics, and therapeutic interventions
Source: Hereditas. 2025 Aug 28;162:175. doi: 10.1186/s41065-025-00545-x (PMC12395852; doi:10.1186/s41065-025-00545-x)

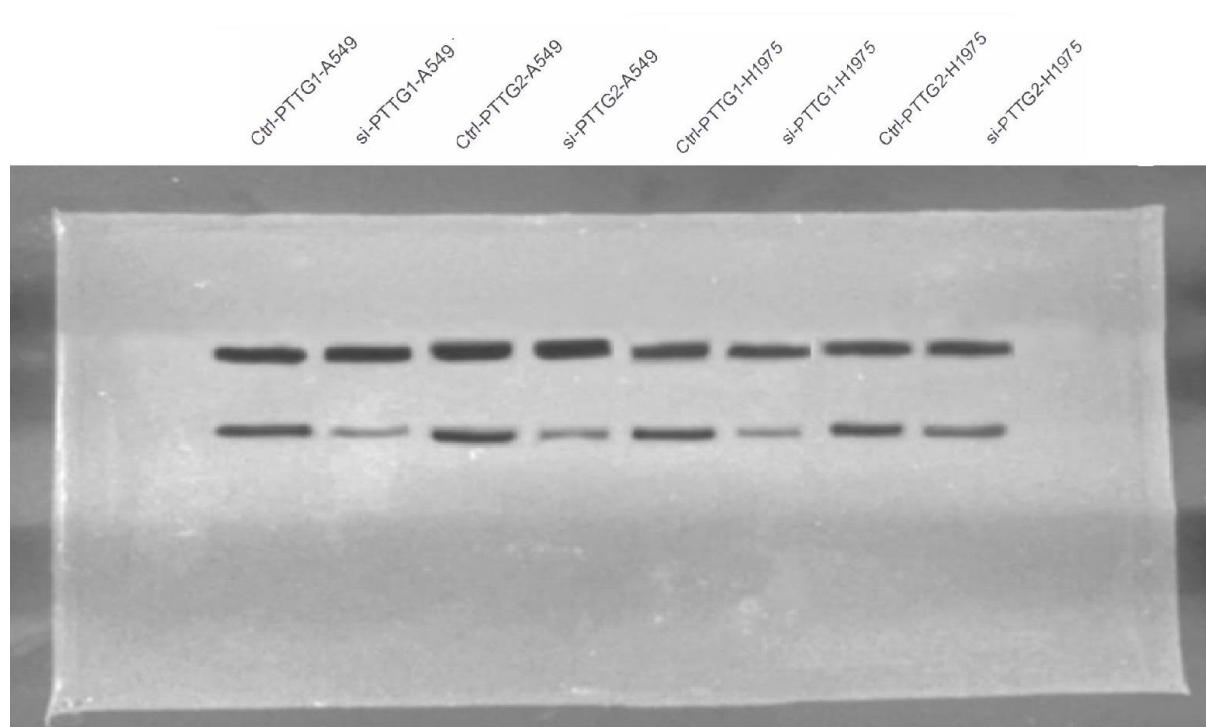

**Supplementary data Figure 1: Uncut Western blot bands of PTTG1, PTTG2, and GAPDH.**

Supplement: Supplementary file 1 — Supplementary Material 1 [file 41065_2025_545_MOESM1_ESM.pdf]
